# Supplementary material for: High-performance deep spiking neural networks with 0.3 spikes per neuron
Source: Nat Commun. 2024 Aug 9;15:6793. doi: 10.1038/s41467-024-51110-5 (PMC11315905; doi:10.1038/s41467-024-51110-5)
Supplement: Supplementary file 1 — Supplementary Information [file 41467_2024_51110_MOESM1_ESM.pdf]

Supplementary Information: High-performance deep spiking neural networks with 0.3 spikes per neuron

Ana Stanojevic[1,2] **Stanisław Woźniak**[1,*] **Guillaume Bellec**[2,3]
Giovanni Cherubini[1] **Angeliki Pantazi**[1] **Wulfram Gerstner**[2,3]

[1] IBM Research Europe – Zurich, Rüschlikon, Switzerland
[2] School of Computer and Communication Science, École Polytechnique Fédérale de Lausanne, Lausanne, Switzerland
[3] School of Life Science, École Polytechnique Fédérale de Lausanne, Lausanne, Switzerland

Supplementary Note 1: Generalization to other neuronal dynamics

Linearization of the double exponential In this paper we solve the vanishing-gradient problem for a spiking neural network with piecewise linear postsynaptic potential, i.e. an input spike at time $t_j^{(n-1)} < t$ causes the following response $a_i^{(n)}(t)$ in neuron i of layer n:

$$a_i^{(n)}(t) = \frac{t - t_j^{(n-1)}}{\tau_c} H(t - t_j^{(n-1)}). \quad (11)$$

However, biologically inspired models in related works [50, 12] often use a double-exponential filter, i.e.:

$$a_i^{(n)}(t) = [1 - \exp(-\frac{t - t_j^{(n-1)}}{\tau_1})]\exp(-\frac{t - t_j^{(n-1)}}{\tau_2})H(t - t_j^{(n-1)}), \quad (12)$$

where τ_1 and τ_2 are time constants and $\tau_2 \geq 2\tau_1 > 0$ (Supplementary Fig. 1).

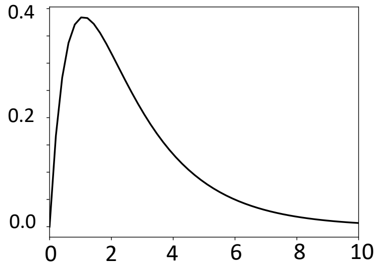

Supplementary Figure 1: An example of $a(t) = [1 - \exp(-\frac{t}{\tau_1})]\exp(-\frac{t}{\tau_2})$ function for $\tau_1 = 1, \tau_2 = 2$.

We notice that in the vicinity of zero, the exponential function can be expressed using Taylor expansion as: $\exp(-\frac{t}{\tau}) = [1 - \frac{t}{\tau} + (1/2)(\frac{t}{\tau})^2 \ldots]$. Therefore the equation for $a_i^{(n)}(t)$ of the double-exponential filter can be approximated around 0 as:

$$a_i^{(n)}(t) = \frac{t - t_j^{(n-1)}}{\tau_1} H(t - t_j^{(n-1)}). \quad (13)$$

To show the relationship between this linearized model we first have to set constant $\tau_c = \tau_1$ in the neuron dynamics in Eq. (1) to match the physically interpretable time constant τ_1. As a result the

*Corresponding author. Email: stw@zurich.ibm.com

potential $V_i^{(n)}$ of neuron i in layer n at time t becomes:

$$V_i^{(n)}(t) = \begin{cases} A_i^{(n)} \dfrac{t - t_{\min}^{(n-1)}}{\tau_1} + \sum_j W_{ij}^{(n)} \dfrac{t - t_j^{(n-1)}}{\tau_1} H(t - t_j^{(n-1)}) & \text{for} \quad t \leq t_{\min}^{(n)} \\ B_i^{(n)} \dfrac{t - t_{\min}^{(n)}}{\tau_1} + V_i^{(n)}(t_{\min}^{(n)}) & \text{for} \quad t_{\min}^{(n)} < t \leq t_{\max}^{(n)} \end{cases} \tag{14}$$

An analogy with the linearized model is therefore possible if the values $A_i^{(n)}$ and $W_{ij}^{(n)}$ represent the rising slopes of post-synaptic potentials for a more biological neuron. The remaining difference with the linearized model arises in the second phase, i.e. the interval $t_{\min}^{(n)} < t \leq t_{\max}^{(n)}$, which does not follow a classical integrate and fire model. To reconcile our two-phase model with a more biologically plausible single-phase integrate and fire model, we can assume that all neurons of layer n that did not spike before $t_{\min}^{(n)}$ are forced to spikes at time $t_{\min}^{(n)}$ (for instance with a strong excitatory input shared for the entire layer). In this way, continuing the first phase after $t_{\min}^{(n)}$ yields the dynamics: $V_i^{(n)}(t) = (A_i^{(n)} + \sum_j W_{ji}) \frac{t - t_{\min}^{(n)}}{\tau_1} + V_i^{(n)}(t_{\min}^{(n)})$ which is equivalent to our two-phase model as long as $B_i^{(n)} = A_i^{(n)} + \sum_j W_{ji}$. This analogy enables the extrapolation of the theoretical conditions for stable gradient descent optimization to the linearized model: the condition $B_i^{(n)} = 1$ yields the constraint $A_i^{(n)} + \sum_j W_{ji} = 1$ in a plausible single phase model. Outside of the linearized setting, we conjecture from our theoretical analysis that the recipe to propagate gradients robustly is generally to cross the threshold with slope 1.

Scaling the interval $[t_{\min}^{(n)}, t_{\max}^{(n)})$ to stay in the linear range In order for the network dynamics to always remain in the linear ramping phase of the double-exponential filter, we need to ensure that the linear approximation given in Eq. (13) is satisfied within the entire coding interval $\max_n [t_{\max}^{(n)} - t_{\min}^{(n-1)})$. As the maximum of the function is reached at $t > \tau_1$ (Supplementary Fig. 1), we require for the implementation of our SNN with a double-exponential model that:

$$\max_n [t_{\max}^{(n)} - t_{\min}^{(n-1)}] < 0.5\tau_1. \tag{15}$$

This is possible if we separate the definition of the interval τ_c of the pixel encoding from the neuron time constant τ_1 (see Supplementary Note 2 for the recursive construction of the intervals $[t_{\max}^{(n)} - t_{\min}^{(n-1)})$). Keeping the notation τ_1 for the neuron time constant and τ_c for the pixel encoding, we can construct our network with an arbitrary scaling factor between them to fulfill Eq. (15).

Example for a CIFAR10 network In order to observe what could be concrete values satisfying the condition given in Eq. (15), let's take as an example the CIFAR10 dataset and VGG16 architecture, already explored in detail in the section on fine-turning for hardware. For the model that was fine-tuned for reduced latency (Fig. 5d), the $[t_{\min}^{(n-1)}, t_{\max}^{(n)})$ interval has a value of around $10\tau_c$. Therefore, the acceptable value for τ_1 is $\tau_1 = 20\tau_c$.

In addition to minor mismatches between the linear and double-exponential postsynaptic potential, caused by the above approximation steps, further mismatches may arise from the heterogeneities of the hardware and other hardware constraints. It is likely that fine-tuning the model, as explained in the main text, would become crucial in this setting.

Supplementary Note 2: Setting $t_{\max}^{(n)}$ and the threshold

Initialization As indicated in the main text, the base threshold $\tilde{\vartheta}_i^{(n)}$ and the parameter $t_{\max}^{(n)}$ are initialized recursively starting from the input interval $[t_{\min}^{(0)}, t_{\max}^{(0)})$. Let us now assume that we have adjusted the base threshold and the maximum firing time $t_{\max}^{(n-1)}$ up to layer $n-1$.

In layer n, the earliest firing time $t_{\min}^{(n)}$ is defined as: $t_{\min}^{(n)} = t_{\max}^{(n-1)}$. At time $t_{\min}^{(n)}$ we evaluate the membrane potential of all neurons in layer n and determine its maximum: $\max_{\mu,i} V_i^{(n)}(t_{\min}^{(n)})$, where the index μ is running over many samples from the training dataset and i iterates over all neurons in

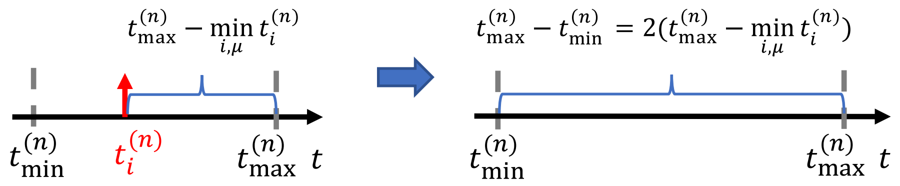

Supplementary Figure 2: **Updating the latest spike time $t_{\max}^{(n)}$ using $\gamma = 2$.** If the minimal spiking time across neurons i and batch inputs μ lies in the first half of the interval $[t_{\min}^{(n)}, t_{\max}^{(n)})$, the interval is extended.

layer n. Since, for all valid mappings, the slope factor $B_i^{(n)}$ of trajectories is positive for $t > t_{\min}^{(n)}$, we define a reference potential in layer n as:

$$\tilde{V}_0^{(n)} = (1+\zeta) \max_{\mu,i} V_i^{(n)}(t_{\min}^{(n)}), \tag{16}$$

where $\zeta > 0$ is a small safety margin. In simulations, we set $\zeta = 0.5$. In case of a reduced latency, $\zeta = 0.5$ is kept for convolutional layers, and otherwise $\zeta = 0$. We choose the latest possible firing time $t_{\max}^{(n)}$ to be

$$t_{\max}^{(n)} \stackrel{\text{def}}{=} t_{\min}^{(n)} + \tau_c \tilde{V}_0^{(n)} / B_0, \tag{17}$$

where B_0 is a reference slope factor of unit value. We then set the base threshold for neuron i in layer n to

$$\tilde{\vartheta}_i^{(n)} \stackrel{\text{def}}{=} B_i^{(n)} \left(\frac{t_{\max}^{(n)} - t_{\min}^{(n)}}{\tau_c} \right). \tag{18}$$

Since the neuron-specific threshold $\vartheta_i^{(n)} \stackrel{\text{def}}{=} \tilde{\vartheta}_i^{(n)} - D_i^{(n)}$ is initialized with a shift parameter $D_i^{(n)} = 0$, the choice in Eqs. (16) – (18) guarantees that, with our initialization of the network parameters, the threshold is reached at a time $t > t_{\min}^{(n)}$ from below.

Note that Eq. (18) defines the base threshold for $t > t_{\min}^{(n)}$. Since for $t < t_{\min}^{(n)}$ the membrane potential trajectories could transiently take a value above $\tilde{\vartheta}_i^{(n)}$, we formally set the threshold for $t < t_{\min}^{(n)}$ to a large value (e.g., $100 \cdot \tilde{\vartheta}_i^{(n)}$) so as to make spiking impossible [41].

For the identity mapping between TTFS-network and ReLU-network, which is the one chosen to avoid the vanishing-gradient problem, the actual slope factor takes a value $B_i^{(n)} = B_0 = 1$. In the context of this mapping, we note that $V_i^{(n)}(t)$ at time $t_{\min}^{(n)}$ has the same value as the activation variable of neuron i in layer n of the equivalent ReLU-network, see Eqs. (1) and (2). Therefore the interval $[t_{\min}^{(n)}, t_{\max}^{(n)})$ is large enough to encode all outputs of layer n in the ReLU-network at initialization (with bias parameter initialized at zero).

Iterative updates during training Throughout training the $t_{\max}^{(n)}$ and the base threshold $\tilde{\vartheta}_i^{(n)}$ are related by Eq. (18). In each iteration the parameters $W_{ij}^{(n)}$ and $D_i^{(n)}$ change. Whenever necessary, the iterative update rule for $t_{\max}^{(n)}$ in Eq. (8) shifts the maximal firing time in a regime with additional safety margin. This influences in turn the threshold $\vartheta_i^{(n)}$ which is recalculated according to Eq. (18).

Additional Remarks (i) In principle, we are free to initialize $t_{\max}^{(n)}$ (or $\tilde{\vartheta}_i^{(n)}$) at arbitrarily high values, much larger than those proposed above. In this case the adaptive rule for $t_{\max}^{(n)}$ (Supplementary Fig. 2) can be omitted. However, the trade-off becomes a very long spiking delay, in particular in networks with many layers.

Both the initialization of the reference potential with a parameter $\zeta > 0$ and the iterative update rule for $t_{\max}^{(n)}$ in Eq. (8) provide a safety margin that leads to spiking delays that could potentially be avoided. However, the $t_{\max}^{(n)}$ used during training does not need to be same as during inference. In order to reduce the classification latency during inference, $t_{\max}^{(n)}$ is recalculated with the fixed parameters found after training such that, in each layer n, the earliest possible spike across all neurons and a representative sample from the training base happens immediately after $t_{\min}^{(n)}$ which in turn leads to a tight value for the threshold via Eq. (18).
